# Generating aldehyde-tagged antibodies with high titers and high formylglycine yields by supplementing culture media with copper(II)

**DOI:** 10.1186/s12896-016-0254-0

**Published:** 2016-02-24

**Authors:** Dona York, Jeanne Baker, Patrick G. Holder, Lesley C. Jones, Penelope M. Drake, Robyn M. Barfield, Gregory T. Bleck, David Rabuka

**Affiliations:** Catalent Pharma Solutions, 5703 Hollis Street, Emeryville, CA 94608 USA; Catalent Pharma Solutions, 726 Heartland Trail, Madison, WI 53717 USA

**Keywords:** Aldehyde tag, formylglycine-generating enzyme, FGE, fGly, SMARTag™, Site-specific, Conjugation, Sntibody-drug conjugate, ADC

## Abstract

**Background:**

The ability to site-specifically conjugate a protein to a payload of interest (e.g., a fluorophore, small molecule pharmacophore, oligonucleotide, or other protein) has found widespread application in basic research and drug development. For example, antibody-drug conjugates represent a class of biotherapeutics that couple the targeting specificity of an antibody with the chemotherapeutic potency of a small molecule drug. While first generation antibody-drug conjugates (ADCs) used random conjugation approaches, next-generation ADCs are employing site-specific conjugation. A facile way to generate site-specific protein conjugates is via the aldehyde tag technology, where a five amino acid consensus sequence (CXPXR) is genetically encoded into the protein of interest at the desired location. During protein expression, the Cys residue within this consensus sequence can be recognized by ectopically-expressed formylglycine generating enzyme (FGE), which converts the Cys to a formylglycine (fGly) residue. The latter bears an aldehyde functional group that serves as a chemical handle for subsequent conjugation.

**Results:**

The yield of Cys conversion to fGly during protein production can be variable and is highly dependent on culture conditions. We set out to achieve consistently high yields by modulating culture conditions to maximize FGE activity within the cell. We recently showed that FGE is a copper-dependent oxidase that binds copper in a stoichiometric fashion and uses it to activate oxygen, driving enzymatic turnover. Building upon that work, here we show that by supplementing cell culture media with copper we can routinely reach high yields of highly converted protein. We demonstrate that cells incorporate copper from the media into FGE, which results in increased specific activity of the enzyme. The amount of copper required is compatible with large scale cell culture, as demonstrated in fed-batch cell cultures with antibody titers of 5 g · L^−1^, specific cellular production rates of 75 pg · cell^−1^ · d^−1^, and fGly conversion yields of 95–98 %.

**Conclusions:**

We describe a process with a high yield of site-specific formylglycine (fGly) generation during monoclonal antibody production in CHO cells. The conversion of Cys to fGly depends upon the activity of FGE, which can be ensured by supplementing the culture media with 50 uM copper(II) sulfate.

**Electronic supplementary material:**

The online version of this article (doi:10.1186/s12896-016-0254-0) contains supplementary material, which is available to authorized users.

## Background

**Fig. 1:**
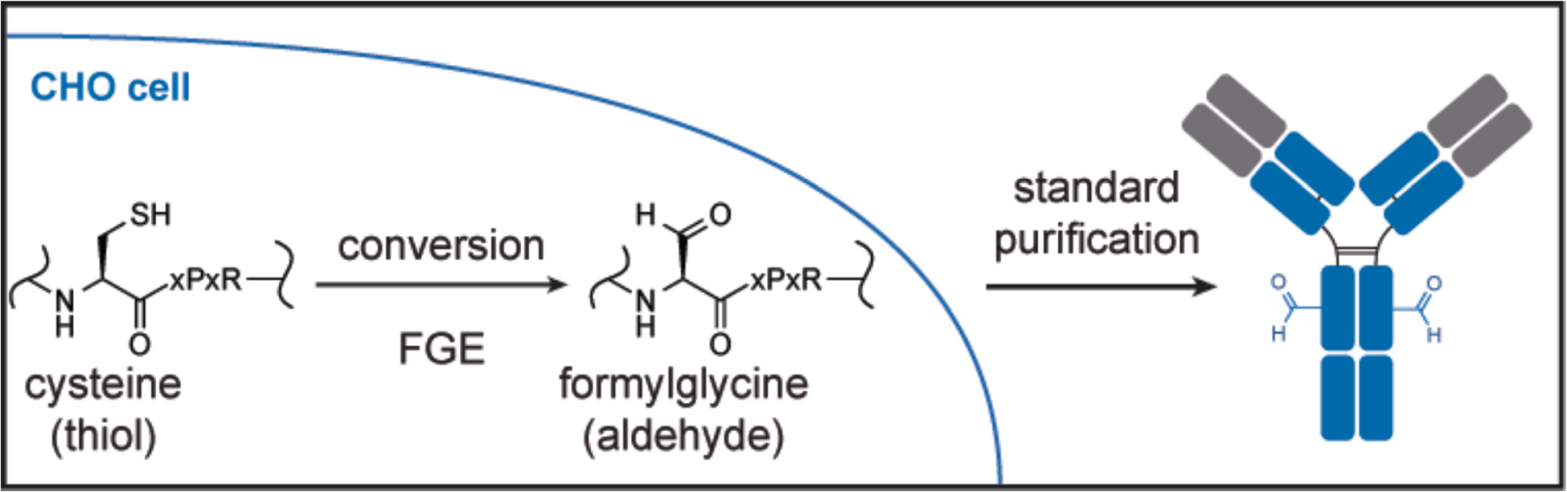
Formylglycine-generating enzyme (FGE) converts the Cys found within in its consensus sequence to an fGly residue. The FGE consensus sequence, CXPXR, where X is any residue except proline, is genetically encoded into a desired location within the target protein of interest, e.g., an antibody. Vectors encoding this “aldehyde-tagged” protein and the FGE enzyme are introduced into a cell; then, during protein production FGE cotranslationally modifies the Cys within the CXPXR sequence, removing the thiol group and replacing it with an aldehyde, resulting in an overall conversion from a Cys to a formylglycine (fGly) residue. The aldehyde group within the fGly serves as a chemical handle that can be used for downstream site-specific bioconjugation

Formylglycine (fGly) is a cotranslational modification of cysteine that can be used as a bioorthogonal chemical handle for site-specific protein conjugation [1, 2]. fGly is installed on a protein in the endoplasmic reticulum (ER) through the action of formylglycine-generating enzyme (FGE). FGE binds to its consensus sequence, CXPXR, where X represents any amino acid except proline, and catalyzes the oxidation of the Cys thiol to an fGly aldehyde (Fig. 1). The endogenous substrates for FGE are sulfatase enzymes that use the fGly residue in their catalytic cycle. However, FGE can also recognize non-native substrate proteins bearing the FGE consensus sequence [3]. This substrate flexibility has been exploited by protein engineers in order to site-specifically modify proteins with a bioorthogonal chemical handle. Using standard molecular biology techniques, the FGE consensus sequence—or “aldehyde tag”—can be inserted at a desired location within a protein of interest. Coexpression of FGE with the aldehyde-tagged protein leads to fGly-containing proteins (Fig. 1). Although proving to be a useful tool [4–10], the yield of Cys to fGly conversion on these recombinant proteins has been inconsistent. For example, when aldehyde-tagged Fc proteins were expressed in CHO cells—which express low levels of hamster FGE—in the absence of exogenous FGE overexpression, the proportion of fGly to Cys in the final product was 28–67 % [3]. The reported fGly yield improved somewhat when FGE was overexpressed in the cells via transient (45–91 %) or stable (62–77 %) transfection [1].

**Fig. 2:**
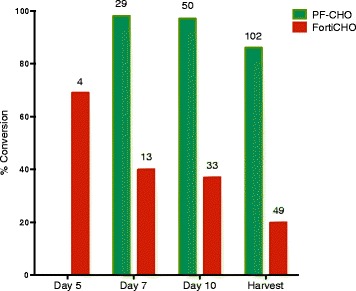
fGly conversion is affected by cell culture media and time in culture. A CHO-S clone (clone 101) stably expressing FGE + KDEL and CT-tagged antibody was cultured in PF-CHO™ or FortiCHO™, fed with 10 % Efficient Feed C on Days 3, 5, and 7, and supplemented with 3 g/L glucose when levels dropped below 3 g/L. Samples of media were taken at the indicated days (harvest was at day 11 and day 14 for FortiCHO™ and PF-CHO™ cultures, respectively). Titers (indicated by the numbers atop bars, mg/L) were assessed by ELISA, and conversion was determined by mass spectrometry. Representative data are shown (*n* =2 experiments)

We use the aldehyde tag in combination with proprietary aldehyde-reactive chemistries as a site-specific conjugation platform called SMARTag™. Our technology aims to enable the large-scale production and commercialization of aldehyde-tagged (and conjugated) proteins, such as antibody-drug conjugates [9, 10]. To this end, we sought to reduce the variability in fGly yield and push the protein yields as high as possible in order to achieve maximum production efficiency. We recently determined that the catalytic mechanism employed by FGE requires a copper cofactor [11]. With that in mind, we explored supplementing cell cultures with copper, and found that addition of 50 μM copper(II) sulfate to the culture media was sufficient to significantly increase fGly yields, even under manufacturing conditions using fed-batch cell cultures with antibody titers of 5 g · L^−1^ and in a 100 L bioreactor run reaching 2.9 g · L^−1^.

## Results and discussion

### Intracellular FGE levels do not correlate with Cys to fGly conversion

As a first step to address the variability of fGly conversion levels, we considered that differences in intracellular FGE levels might affect conversion yields. Wild-type human FGE is partially retained in the ER through interactions of its *N*-terminal region (called the *N*-terminal extension, NTE) with endoplasmic reticulum resident protein 44 (ERp44) [12]. This interaction can be abrogated by furin-mediated proteolytic cleavage at an internal site, allowing the remaining catalytically-active core enzyme to be secreted [11]. With this in mind, we took two approaches towards retaining FGE inside the cell. One was to delete the furin cleavage site, leaving FGE intact and able to retain its interactions with ERp44. The other was to incorporate an alternative ER-retention signal—a KDEL sequence—at FGE’s *C*-terminus. In some iterations, both modifications were included in the same construct.

Plasmids encoding these various forms of FGE, including wild-type, furin site-deleted (DFur), +KDEL, and DFur + KDEL, were used to make CHO-S clonal cell lines stably expressing the FGE isoforms under the control of the human EF1α promoter. Then, from these lines we selected FGE clones with varying levels of intracellular FGE as determined by flow cytometry and enzyme-linked immunosorbent assay (ELISA)-based experiments (Additional file 1: Figure S1). Next, we tested the efficiency of conversion on transiently-expressed antibodies in these clones. Furthermore, we also generated stable pools of antibody-expressing cells by transfecting these FGE clones. Together, the transient transfections and stable pools covered two antibody backbones and two aldehyde tags placed at different locations—at the *C*-terminus of the heavy chain (CT) and in the hinge region. Although the antibody titers from these cultures were low, conversion analyses demonstrated that there was no correlation between conversion efficiency and intracellular FGE levels, or forms of FGE, under these conditions (Table 1).

**Table 1:** FGE form does not correlate with conversion yield

			Transient Ab 1, C_H_-CT		Transient Ab 2, Hinge		Stable Ab 1, C_H_-CT		Stable Ab 2, C_H_-CT		Stable Ab 2, Hinge	
Clone	FGE form^a^	FGE levels	Titers (mg/L)	Conversion	Titers (mg/L)	Conversion	Titers (mg/L)	Conversion	Titers (mg/L)	Conversion	Titers (mg/L)	Conversion
395-12	WT	Low	11.8	96 %	10.5	81 %	ND	ND	2.1	99 %	1.6	99 %
395-12	WT	Low	3.7	90 %	6.6	69 %	ND	ND	1.6	98 %	ND	ND
408-102	∆Fur + KDEL	High	11.5	95 %	9.9	81 %	ND	ND	1.1	98 %	1.2	90 %
409-107	∆Fur + KDEL	Low	1.4	83 %	6.8	69 %	2	97 %	1.1	97 %	ND	ND
407-118	+KDEL	High	11.8	97 %	ND	ND	4.7	97 %	0.9	98 %	1.3	99 %
406-127	∆Fur	Med	1.9	80 %	7.4	54 %	7.8	92 %	1.5	94 %	ND	ND
409-35	∆Fur + KDEL	Med	ND	ND	6.5	68 %	1.7	92 %	2	98 %	ND	ND

^a^∆Fur, deleted furin cleavage site

### fGly conversion is affected by cell culture media and time in culture

From this work, we selected a stable CHO-S clone expressing FGE + KDEL and CT-tagged antibody 2 (clone 101, Table 1) to test the effect of media on fGly yield. As a starting point, we cultured the cells in either CD FortiCHO™ or PF-CHO™ LS, and measured titer, viability, cell density, and conversion to fGly. We observed that the media had a pronounced effect on the fGly yield (Fig. 2). Cells cultured in PF-CHO™ LS yielded highly converted antibody throughout the production run with 98 % fGly at day 7 and 86 % fGly at day 14. By contrast, cells cultured in CD FortiCHO™ produced antibody that contained a decreasing amount of fGly as time in culture proceeded. While antibody from this culture contained 70 % fGly at day 5, by day 11 the proportion of fGly had dropped to 20 %. Meanwhile, the viability of the cells over time was similar under both conditions, but the titers were approximately two-fold higher in the PF-CHO™ LS media, and cell density was two- to three-fold higher in the CD FortiCHO™ media (Table 2).

**Table 2:** Conversion to fGly is highly dependent on cell culture conditions

Media	Culture day	Density (million/mL)	Viability	Titer (mg/L)	Conversion
PF-CHO	Day 7	2.8	90 %	29.4	98 %
	Day 10	4.5	95 %	49.7	96 %
	Day 14	3.4	80 %	101.7	86 %
FortiCHO	Day 5	>10	99 %	4.4	70 %
	Day 7	17	99 %	12.6	42 %
	Day 10	10	80 %	33.2	36 %
	Day 11	5.3	66 %	48.7	20 %

### Addition of copper(II) sulfate to cell culture media increases conversion yields

**Fig. 3:**
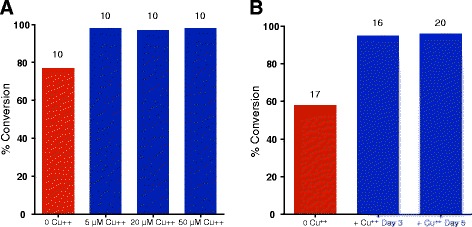
fGly conversion proceeds efficiently in cells cultured in the presence of 5–50 μM copper(II) sulfate. CHO-S clone 101 stably expressing FGE + KDEL and CT-tagged antibody was cultured in FortiCHO™ +/- the indicated concentrations of copper(II) sulfate added on day 0 (**a**), or with 50 μM copper(II) sulfate added on days 3 or 5 (**b**). Cultures were fed with 10 % Efficient Feed C on Days 3, 5, and 7. Media was harvested at day 10 (**a**) or day 12 (**b**); titers (indicated by the numbers atop bars, mg/L) were assessed by ELISA, and conversion was determined by mass spectrometry. *n* = 7 experiments for 0 and 50 μM copper(II) sulfate added on day 0; other time points and copper(II) sulfate concentrations were tested once

The dependence of fGly yields on cell culture conditions suggested that a media component might be limiting. We recently determined that the catalytic mechanism employed by FGE requires a copper cofactor [11]. Therefore, the cells require a stoichiometric amount of copper to match FGE enzyme levels in order to ensure FGE activity. The disparity observed in the conversion levels of antibodies produced in PF-CHO™ LS and CD FortiCHO™ media could reflect differing amounts of copper contained in those proprietary media (for which the components are not disclosed). Furthermore, the decreasing conversion yields observed over time in culture—most strongly exemplified in the CD FortiCHO™ cultures—may reflect depletion of copper in the media through cell growth and metabolism. In order to test this theory, we cultured clone 101 in CD FortiCHO™ media +/- supplementation with 5, 20, or 50 μM copper(II) sulfate added on day 0. Media was harvested on day 10 and antibodies were analyzed for conversion (Fig. 3a). The results showed that addition of 5 μM or more copper(II) sulfate led to fGly yields of ≥ 96 % as compared to a 77 % yield in cells that were not supplemented with copper.

In order to test the dependence of timing on this effect, we cultured clone 101 in CD FortiCHO™ media +/- supplementation with 50 μM copper(II) sulfate added on day 3 or day 5. Media was harvested on day 12 and antibodies were analyzed for conversion (Fig. 3b). The results showed that addition of copper at either time point gave similar results as addition at day 0; namely, fGly yields ≥ 95 % as compared to a 58 % yield in cells that were not supplemented with copper.

In order to test the specificity of the observed copper effect on fGly yields, we tried substituting various other metal ions that are common enzyme cofactors, including iron sulfate, magnesium chloride, and zinc chloride (Additional file 1: Figure S2)_._ None of these metals improved the fGly yield over media alone. This result was consistent with our observations of purified FGE, which showed that only copper, but not these other cofactors, could increase the enzyme’s specific activity [11].

### Addition of copper(II) sulfate to cell culture media results in FGE copper loading and holoenzyme formation

**Fig. 4:**
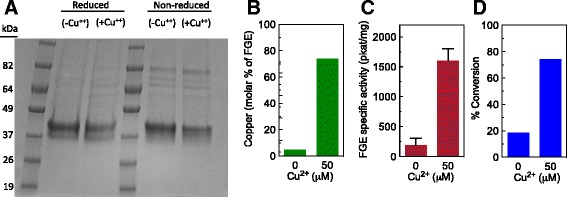
FGE purified from copper(II) sulfate-supplemented cells contains copper and has enhanced specific activity. A gene encoding FGE containing a His_6_ affinity tag was cotransfected with CT-tagged antibody into Expi293™ cells. Cells were cultured +/- supplementation with 50 μM copper(II) sulfate. On day 4, antibody was harvested. Then, the cells were washed to remove excess media (and free copper), lysed, and H_6_-*Hs*-FGE was purified from the cell lysate using metal affinity chromatography. Purified FGE was analyzed by reducing and non-reducing SDS-PAGE (a). Then, the purified H_6_-*Hs*-FGE samples were assayed for copper content—by ICP-MS (b)—and for specific activity—via an HPLC-based assay on a peptide substrate (c). Conversion of the antibody was determined by mass spectrometry (d). The experiment was repeated four times with similar results; error bars in C represent standard deviation (*n* = 4). ICP-MS copper analysis (b) and conversion analysis of the cotransfected antibody were performed once

Transport and delivery of copper is a tightly regulated process in eukaryotes [13]. By one estimate, there is no unbound (free) intracellular copper [14]. Therefore, the addition of copper to culture media did not guarantee that it would be incorporated into the FGE enzyme. To test this, we prepared a variant of human FGE containing a His_6_ affinity tag (H_6_-*Hs*-FGE). Transient cotransfection of vectors containing H_6_-*Hs*-FGE and the CT-tagged antibody was performed in Expi293™ cells +/- supplementation of 50 *μ*M copper(II) sulfate in the cell culture media. After 4 days in culture, the cells were washed to remove excess media (and free copper), lysed, and H_6_-*Hs*-FGE was purified from the cell lysate using metal affinity chromatography (Fig. 4a). After purification, H_6_-*Hs*-FGE was assayed for copper content and specific activity (Fig. 4b and c). Inductively coupled plasma mass spectrometry (ICP-MS) analysis of the purified enzymes demonstrated that the enzyme purified from copper-treated cells contained more copper (0.74 mol/FGE, 74 % holo enzyme) as compared to the enzyme purified from untreated cells (0.03 mol/FGE, 3.0 % holo enzyme). Accordingly, we found that the FGE isolated from cells grown in copper-containing media was significantly more active [1604 ± 196 picokatal (pkat) per mg] than that isolated from untreated cells (190 ± 116 pkat/mg). Finally, we measured the fGly content in the purified antibody, which also correlated with the presence (74 % fGly) or absence (19 % fGly) of the copper supplement (Fig. 4d). Together, these data demonstrated that addition of copper(II) sulfate to the cell culture media was sufficient to provide FGE with the required copper cofactor, which then allowed the enzyme to efficiently convert Cys to fGly during protein production. Interestingly, this copper loading does not occur efficiently when FGE is produced in *E. coli* [11]. Copper supplementation of bacterial cultures does result in a modest improvement in holoenzyme formation (data not shown), but to a much lesser extent than that observed in mammalian cells. Insect cells cultures are also efficient at holoenyzme formation [11].

### High conversion and titers are observed in transiently transfected cultures supplemented with copper(II) sulfate

**Fig. 5:**
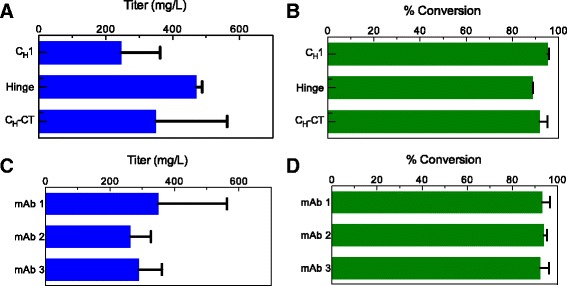
Aldehyde tag conversion is independent of tag location and antibody type. The aldehyde tag was incorporated at various positions across an antibody heavy chain—at the C_H_1, hinge, or heavy chain *C*-terminus (C_H_-CT). Cotransfection of vectors encoding FGE and the relevant heavy and lights chains in Expi293™ cells enabled transient production of fGly-containing antibodies in good titer (**a**) and with high conversion (**b**). By comparison, when untagged antibody was expressed following the same procedure, the titer was 223 mg/L. Installation of the aldehyde tag at the C_H_-CT of three different IgG1-type antibodies followed by coexpression with FGE in Expi293™ cells resulted in the same high titers (**c**) and conversion (**d**). All panels, *n* = 3; error bars represent standard deviation

Because the previous experiments were conducted using stable FGE clones, next we tested whether conversion could proceed efficiently in a fully-transient expression system (Fig. 5). For this purpose, we used Expi293F™ cells cotransfected with three vectors, one each encoding FGE, the antibody light chain, and the antibody heavy chain. Furthermore, we asked whether FGE could convert aldehyde tags installed at any of several locations on the same antibody, or at the same location on multiple human IgG1 antibodies. With respect to tag placement, we tested three variants of an antibody with the aldehyde tag installed in either the C_H_1, the hinge, or at the CT. The transient titers of these antibodies ranged from 157 to 578 mg/L (Fig. 5a), and the conversion of Cys to fGly was consistently high (88–97 %, Fig. 5b). With respect to antibodies with different variable regions, the installation of the aldehyde tag at the same site (CT) across three different IgG1 antibodies resulted in similar titers and high conversion (Fig. 5c and d).

## GPEx® system-derived clones stably expressing both FGE and aldehyde-tagged antibody yield high titers and high conversion

**Fig. 6:**
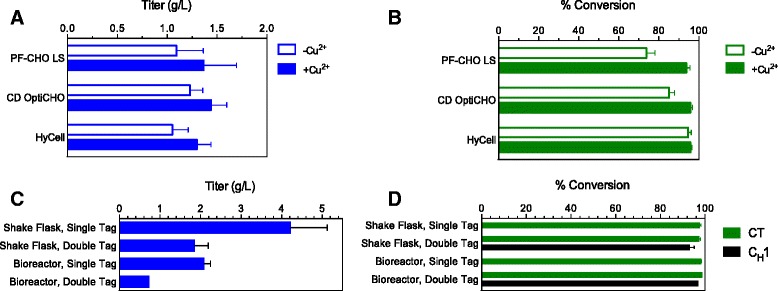
High fGly conversion yields can be obtained from stably transduced cell lines under high titer cell culture conditions. Stable transduction of DNA encoding FGE, antibody light chain, and antibody heavy chain bearing one (CT only) or two (C_H_1 and CT) aldehyde tags was performed with viral retrovector transduction into CHO cells (GPEx® technology). The resulting stable pools were cultured in three types of media +/- supplementation with copper(II) sulfate (*n* = 3). Titers (**a**) and fGly conversion (**b**) of CT-tagged antibodies were assessed. Then, the stable pools were cloned by limiting dilution and clone performance was assessed in fed batch cultures (**c** and **d**; shake flask, *n* = 5 clones; bioreactor, *n* = 3 clones single tag, *n* = 1 clone double tag). The top performing stable single-tagged clonal cell line produced antibody in very high titer (5.2 g/L) with 98 % conversion of Cys to fGly (**c** and **d**). The specific productivity (75 pg/cell/d) of this top clone demonstrates the capabilities of the GPEx® technology for efficient production of highly converted antibody. The generation of fGly in single- and doubly-tagged antibodies scaled successfully to bioreactors (2 L cultures; **c** and **d**). Error bars indicate standard deviation

Bolstered by these positive results in transient cultures, we next tested stable cell lines that could eventually be expanded into a manufacturing process. A retrovector transduction (GPEx® technology; [15]) of CHO cells was performed to incorporate human FGE into cellular DNA, generating stable pools from which clones were selected. Stable expression of human FGE in the clones was confirmed by flow cytometry. Then, the FGE clones were transduced to generate stable pools of antibody expressing cells. These cells were tested for antibody titer and fGly conversion during culture in three common media types, for which the copper content is not disclosed: PF-CHO™ LS, CD OptiCHO™, and HyCell™ (Fig. 6). Without copper(II) sulfate supplementation, we observed good titers, but variable conversion yields in antibodies produced in these media. By contrast, addition of copper to the cultures led to consistently high fGly content without decreasing titers. In the case of PF-CHO™ LS, the yield of fGly in the purified antibody increased from 74 ± 4 % (*n* = 3) to 94 ± 2 % (*n* = 3).

**Fig. 7:**
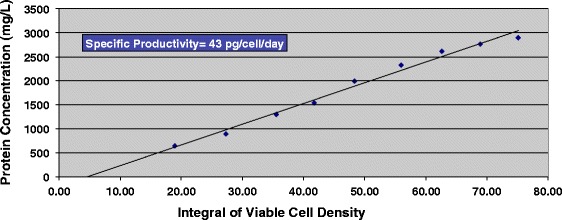
A GPEx® clonal cell line expressing CT-tagged antibody afforded high fGly conversion and high titers in a 100 L bioreactor production run. Cells were cultured in HyCell™ media supplemented with 50 μM copper(II) sulfate. The protein concentration and integral of viable cell density is shown. Cultures were terminated when viabilities were ≤ 80 %. Titers of 2.9 g/L were reached. The cells demonstrated a specific productivity of 43 pg/cell/d, and the resulting antibody had fGly conversion yields of 96.4 %

Subcloning of these stable pools by limiting dilution afforded cell lines that produced high amounts of protein (4.2 ± 0.9 g/L, *n* = 5) in fed batch shake flask cultures (Fig. 6c). More importantly, the conversion yield remained extremely high (98 ± 1 %, *n* = 5) even with the increased titers and productivity (Fig. 6d). We also generated clonal cell lines producing antibody containing two aldehyde tag sites per heavy chain (C_H_1 and CT, or “double tag”), resulting in four fGly sites per antibody. Doubling the number of sites was well-tolerated by the FGE-producing cell lines. For the top performing doubly-tagged clones, the yields of fGly were 93.2 % ± 1.9 in the C_H_1 and 97.4 % ± 0.5 at the CT (*n* = 5); the antibody titers were 1.9 ± 0.3 g/L (*n* = 5). The clonal cell line productions of singly- and doubly-tagged antbodies were successfully scaled to 2 L bioreactors (Fig. 6c and d). The highest antibody-producing clone, with a titer of 5.2 g/L, had a specific productivity (Q_p_) of 75 pg/cell/d. To confirm that these cell lines and processes are scalable for development of clinical and commercial products, a 100 L production run was performed. As expected the results were similar to the 2 L production with titers reaching 2.9 g/L, specific productivity of 43 pg/cell/d, and fGly conversion yields of 96.4 % (Fig. 7).

## Conclusions

The incorporation of fGly has been leveraged for the transport and display of a wide range of payloads, including DNA for protein scaffolding [4]; chromophores for probing protein dynamics [5]; anchors for solid substrates [6]; immobilization of viruses [7] and enzymes [8]; and the site-specific attachment of cytotoxic drugs for the generation of antibody–drug conjugates (ADCs). With site-specific conjugation, it is now possible to build structure activity relationships for ADCs that correlate payload placement on the antibody with efficacy, pharmacokinetics, and conjugate stability [9, 10, 16].

In an effort to scale up production of fGly-containing antibody for preclinical evaluation of next-generation ADCs, we designed a method to generate a high yield of this modification during antibody production. Our results highlight the robust generation of fGly by FGE in vivo. The enzyme is able to maintain a rate of turnover that matches antibody production for the duration of cell culture as long as it has access to sufficient amounts of copper. Perhaps most importantly, the addition of 50 *μ*M copper(II) sulfate to the media has no obvious deleterious effects on cell growth, cell density, protein titer, or culture duration. However, considering what is known about the impact of copper on antibody quality, including the production of basic variants [17], we plan to continue to refine the copper requirements during antibody production. As a first step towards establishing reproducible conversion in the context of a high titer antibody production, the simple solution outlined herein enables the generation of antibodies containing aldehyde tags in a process that is compatible with industrial scale production.

## Methods

### DNA construction of pEF1α-Hs-FGE variants

A pcDNA™3.1 vector encoding for wild-type human FGE (wt-*Hs*-FGE) was available from a previous study [3]. *Hs*-FGE UniProt accession: Q8NBK3; PDB 1Y1E. The FGE gene was amplified by PCR using a primer encoding for the kozak consensus sequence and cloned into pcDNA™3.1(-) hygro to generate pcDNA™3.1-hygro-kozak-FGE flanked by NruI and NheI restriction sites. Subsequently, the EF1α promoter was amplified from the pEF1α-IRES vector (Clontech) by PCR using primers containing NruI/NheI sites. Both the FGE vector and the EF1α PCR product were digested with NruI and NheI and ligated with the Rapid DNA Ligation Kit (Thermo/Life Technologies) to generate WT pEF-FGE-hygro, which was amplified in XL-10 gold cells (Agilent) and purified according to the manufacturer’s instructions. Quikchange Mutagenesis (Agilent) was used to mutate the furin cleavage site (HRYSRE to HRYSGE) and/or add the KDEL retention signal to the C-terminus of FGE to generate Δfurin, +KDEL, and Δfurin + KDEL versions of FGE.

### Transfection of CHO-S cells with pEF-FGE variants and establishment of clonal cell lines

CHO-S cells were transfected with the pEF-FGE-hygro variants described above using FreeStyle™ Max transfection reagent in OptiPro™ serum-free media following the manufacturer’s protocol. After overnight recovery of growth, the cells were pelleted and grown in PF-CHO™ LS (GE Healthcare) under hygromycin selection (400 μg/mL). After 8 d of selection, the cell viability was 10 %. Continued passaging for 14 d allowed recovery of stable pools to 99 + % cell viability (CHO-S-FGE-pool). Then, stable CHO-S-FGE-pools were cloned by limiting dilution in 96-well plates using semi-solid ClonaCell™ media (STEMCELL Technologies). Surviving clones were expanded to six well plates for selection and characterization. The final clones were propagated in CD FortiCHO™ media with 125 μg/mL hygromycin selection.

### Transient transfection of CHO-S-FGE cells with C_*H*_CT-antibody and establishment of clonal cell lines

Two vectors encoding for the target antibody heavy (HC) and light chains (LC) were generated using standard molecular biology techniques from a vector encoding the pCF promoter (the CMV promoter with the adenovirus tripartite leader). CHO-S-FGE cells expressing different FGE variants were transfected with the vectors encoding HC and LC using FreeStyle™ Max transfection reagent in OptiPro™ serum-free media following the manufacturer’s protocol. For transient protein production, cells were cultured under standard conditions for 5 days and antibody was harvested using Protein A. To generate stable clonal cell lines of FGE and antibody expressing cells, transfections were performed as described, then after overnight recovery of growth, the cells were pelleted and grown in PF-CHO™ LS® under both hygromycin (250 μg/mL) and G418 (500 μg/mL) selection. After day 6, the cell viability had decreased to 50 %. Continued passaging under selection for 14 days allowed recovery of the stable pools to 99 + % cell viability (CHO-S-FGE-antibody-pool). The stable CHO-S-FGE-antibody-pools were cloned by limiting dilution in 96-well plates using semi-solid ClonaCell™ media (STEMCELL Technologies). Surviving clones were expanded to six well plates for selection and characterization. The final clones were propagated in CD FortiCHO™ media (Thermo Fisher) with hygromycin (125 μg/mL) and G418 (250 μg/mL).

### Fed batch production of antibody from CHO-1

CHO-1 was used to produce antibodies under a variety of experimental conditions, all of which included the antibiotics described for CHO-1 propagation. In brief, CHO-1 was seeded into 30–120 mL of fresh media (typically CD FortiCHO™, PF-CHO™ LS, or HyCell™) at 0.5x10^6^ cells/mL. Copper(II) sulfate (when used) was added on day 0 as a 1000x stock solution in water. The cell culture was fed with supplements on day 0, and with glucose whenever the level was below 2 g/L.

### DNA construction of His6-Hs-FGE in pcDNA3.1(-)myc-His

Wild-type *H. sapiens* FGE (SUMF1, NCBI 285362) was amplified from a pcDNA 3.1 construct available from a previous study [3]. The insert and pcDNA 3.1(-)myc-His (Invitrogen) were digested with EcoRI and HindIII and ligated following the manufacturer’s instructions.

### Transient coexpression of His6-Hs-FGE and C_*H*_CT-antibody

The expression constructs for FGE and the antibody heavy and light chains were introduced into Expi293F™ cells at a 1.6:2:3 ratio (FGE:HC:LC) following the manufacturer’s instructions. Cells were cultured in Expi293™ media with enhancers as specified by the manufacturer. Copper(II) sulfate was added as a 1000x stock solution on the day of transfection. The media and cells were harvested after 4 d. Cells were removed by centrifugation. Antibodies were purified from the conditioned medium using Protein A chromatography with MabSelect SuRe™ resin (GE Healthcare).

### Purification of H_6_-Hs-FGE from Expi293F*™* cells

The Expi293F™ cell pellet was resuspended in lysis buffer (25 mM triethanolamine, 300 mM NaCl, 1 % triton X-100, protease inhibitors). This solution was loaded onto Ni-NTA superflow (Qiagen). The resin was washed with 10 column volumes of wash buffer (25 mM triethanolamine [TEAM], 250 mM NaCl, 5 mM calcium acetate, 10 mM imidazole, pH 8). The enzyme was then removed from the column with elution buffer (25 mM TEAM, 5 mM calcium acetate, 300 mM imidazole, pH 8). Fractions containing protein as determined by protein assay (Bio-Rad) were pooled and loaded onto a Sephadex® G-25 column equilibrated with assay buffer, eluted, and concentrated by centrifugal ultrafiltration.

#### DNA construction and cloning for retrovector production

The FGE gene sequence (SUMF1, NCBI 285362) containing a *C*-terminal KDEL sequence was cloned into the Catalent’s GPEx® expression vector containing the Neomycin-resistant gene. The heavy and light chain antibody gene sequences were codon optimized for CHO cell expression and a proprietary signal peptide sequence was added to the coding sequences. Both DNA sequences of antibody heavy and light chains were cloned into Catalent’s GPEx® expression vectors.

#### Retrovector production for stable transduction

The expression constructs encoding FGE, LC, or HC were introduced into the 293 cells. These cells originate from a fully characterized HEK 293 cell line that constitutively produces the MLV *gag*, *pro*, and *pol*. This replication incompetent system produces high titer retrovector that is concentrated by ultracentrifugation and used for cell transductions.

#### Transduction of GCHO Cells with Retrovector

The GCHO FGE cell line was made by performing three rounds of transduction (multiplicity of > 1000 retrovector particles/cell) of the GPEx® Chinese Hamster Ovary (GCHO) parental cell line with retrovector made from the gene construct developed to express FGE. The GCHO antibody cell line was made by performing multiple rounds of transduction (multiplicity of >1000 retrovector particles/cell) of the GPEx® Chinese Hamster Ovary (GCHO) FGE parental cell line with retrovector made from the gene construct developed to express the antibody LC and HC. Five independent transductions were performed: two LC and three HC.

#### Fed batch production of antibody from the pooled population of cells

Post-transduction, the pooled population of GCHO-Antibody cells was scaled up for productivity in fed batch studies in duplicate 2.8 L shake flasks. Each shake flask was seeded with 300,000 viable cells per mL in a 1400 mL working volume of either PF-CHO™ LS, CD OptiCHO™, or HyCell™ media and incubated in a humidified (70–80 %) shaking incubator at 90 rpm with 5 % CO_2_ at 37 °C. Cultures were fed based on viable cell density and glucose levels. Cultures were terminated when viabilities were ≤ 50 %. Antibody expression was determined by Protein A HPLC using a generic IgG standard.

### Establishment of clonal cell lines from transduced GCHO cells

Limited clonal dilution in 96 well plates was performed on the pooled population of GCHO-Antibody cells. From over 400 clones, the top 20 clones based on protein titer (ELISA using a generic IgG standard) were expanded and analyzed in an enhanced fed batch productivity study.

### Fed batch production of antibody from clonal cell lines (shake flask)

The top twenty clones were selected based on antibody titer from a 96 well plate. The selected clonal cell lines were tested for productivity in an enhanced fed batch overgrowth study. For each clone, duplicate T75 shake flasks were seeded with 300,000 viable cells per mL in 60 mL working volume of HyClone™ HyCell™ media (GE Healthcare) and incubated in a humidified (70–80 %) shaking incubator at 120 rpm with 5 % CO_2_ at an initial temperature of 37 °C. Cultures were fed on designated days. A temperature shift was also performed on Day 4. Cultures were terminated when viabilities were ≤ 50 %. Antibody expression was determined by Protein A HPLC using a generic IgG standard. After production, antibodies were purified from the conditioned medium using Protein A chromatography with MabSelect SuRe™ resin (GE Healthcare).

### Fed batch production of antibody from clonal cell lines (bioreactor)

The clonal cell lines were tested for productivity in an enhanced fed batch overgrowth study in 2 L bioreactors. For each clonal cell line a single 2 L Sartorius/Braun double-walled glass vessel was seeded with 300,000 viable cells per mL in 1400 mL working volume of HyCell™ media (HyClone™). The operating parameters of 2 L bioreactors, controlled by Finesse units, were as follows: initial temperature of 37 °C, dissolved oxygen (DO) of 50 % and pH 7.0 (upper deadband of 0.60, lower deadband of 0.20). Cultures were fed on designated days. A temperature shift was also performed on Day 5. Cultures were terminated when viabilities were ≤ 80 %. Antibody expression was determined by Protein A HPLC using a generic IgG standard. After production, antibodies were purified from the conditioned medium using Protein A chromatography with MabSelect SuRe™ resin (GE Healthcare).

#### Flow cytometry

For each sample, 1 million cells were centrifuged at 300 × g. The medium was removed by aspiration and the cells were washed with phosphate buffered saline (PBS) + 1 % bovine serum albumin (BSA) and transferred to a 96 well plate. The PBS was removed by aspiration, and the cells were fixed with 100 μl ice cold methanol on ice for 15 min. The cells were again centrifuged at 300 × g and gently washed with ice cold PBS + 1 % BSA. The cells were then gently resuspended in 100 μl of the primary antibody solution, a 1:100 dilution of mouse anti-human/mouse SUMF1 IgG (R&D Systems) in PBS + 1 % BSA. The cells were then incubated for 1 h at RT with shaking. After, the cells were washed twice with PBS + 1 % BSA, and then resuspended in the secondary Ab solution, a 1:100 dilution of phycoerythrin-conjugated anti-mouse IgG in PBS + 1 % BSA. The cells were then incubated for 1 h at RT with shaking in the dark. Finally, the cells were washed twice with PBS + 1 % BSA and analyzed on a BD FACSCanto™ Flow Cytometer.

#### Quantification of fGly content in an antibody

Determination of fGly content was performed using methods previously described [11]. In brief, fGly content was determined by liquid chromatography-multiple reaction monitoring/mass spectrometry (LC-MRM/MS) after antibody reduction with DTT, followed by proteolysis and chemical modification of Cys (iodoacetamide) or fGly (methoxylamine). Mass Spectrometry data were collected on a 4000 QTRAP® mass spectrometer (AB Sciex) with an 1100 series HPLC (Agilent). Chromatography was performed on a Jupiter™ 150 × 1.0 mm C18 column (Phenomenex) enclosed in a butterfly column heater set to 65 °C with a PST-CHC controller (Phoenix S&T). Calculation of LC-MRM/MS transition masses and integration of the resulting data was performed with Skyline 2.6 [18]. The quantity of each species was integrated and fractions of the total were calculated.

#### Quantification of FGE specific activity

The method for determining FGE specific activity was previously reported [11]; in brief, a synthetic peptide, encoding the consensus sequence for FGE was used in vitro as a substrate for the enzyme. The peptide substrate was made by New England Peptide, Inc. on solid phase and purified to ≥95 %. After treatment of the peptide with FGE, the conversion of Cys to fGly was measured by separating the two forms of the peptide by reversed-phase high performance liquid chromatography (RP-HPLC) and integrating the peak areas for each species. RP-HPLC was performed on an 1100/1200 series instrument (Agilent Technologies) controlled with Agilent OpenLAB CDS Chemstation Edition. The instrument consisted of an in-line solvent degasser, analytical quaternary pump, vial auotsampler, thermostatted column compartment, and diode array detector. Chromatography was achieved on an Aeris™ core-shell 250 × 2.1 mm XB-C18 Widepore column (Phenomenex, Inc.).

#### Absorption spectroscopy

UV and visible absorption spectra were recorded on a NanoDrop™ 2000 (Thermo Scientific) spectrophotometer with manufacturer-supplied software. Spectrometers were calibrated with NIST-traceable potassium dichromate standards for photometric accuracy (Starna Scientific).

#### ICP-MS

Inductively-coupled plasma mass spectrometry (ICP-MS) was performed by the Catalent Center for Excellence in Analytical Services (Morrisville, NC). The ratio of copper was calculated as a mol ratio based upon protein concentrations measured from 280 nm absorption intensity of enzyme stock solutions.

#### Computing and software

All instrument data collection workstations were operated with PCs and instrument control software as provided by the manufacturers. Linear and nonlinear regression was performed using Prism® 6.0e (GraphPad). Integration of HPLC chromatograms was performed using OpenLAB CDS Chemstation Edition (Agilent). Calculation of LC-MRM/MS transition masses and integration of the resulting data was performed with Skyline 2.6 (MacCoss Lab, University of Washington). Statistical calculations were performed with Microsoft Excel.

#### Statistics

When displayed on bar graphs, measurements of uncertainty represent the standard deviation. Area under the curve (AUC) for HPLC runs was calculated by the Chemstation software using the “new exponential” algorithm and setting slope sensitivity of 1 mAU. Kinetic parameters were determined from nonlinear regression of [substrate] v. initial velocity using GraphPad Prism®.

### Ethics approval

Not applicable.

### Consent for publication

Not applicable.

## Additional file

Additional file 1: Table S1. FGE isolated from Expi293™ cell culture contains both copper and calcium as measured by ICP-MS. **Figure S1.** Intracellular FGE levels can be determined by flow cytometry or ELISA. **Figure S2.** The effect of copper(II) sulfate addition on conversion is not replicated by the addition of other metal cofactors. (DOCX 641 kb)

## Abbreviations

ADC: Antibody-drug conjugate
AUC: area under the curve
BSA: bovine serum albumin
C_H_-CT: *C*-terminally-tagged antibody heavy chain
CT: *C*-terminal
DFur: furin site-deleted
ELISA: enzyme-linked immunosorbent assay
ERp44: endoplasmic reticulum resident protein 44
FGE: formylglycine-generating enzyme
fGly: formylglycine
H_6_-*Hs*-FGE: human FGE containing a His_6_ affinity tag
HC: antibody heavy chain
ICP-MS: inductively coupled plasma mass spectrometry
LC: antibody light chain
LC-MRM/MS: liquid chromatography-multiple reaction monitoring/mass spectrometry
PBS: phosphate buffered saline
