# Supplementary material for: Generating aldehyde-tagged antibodies with high titers and high formylglycine yields by supplementing culture media with copper(II)
Source: BMC Biotechnol. 2016 Feb 24;16:23. doi: 10.1186/s12896-016-0254-0 (PMC4766608; doi:10.1186/s12896-016-0254-0)
Supplement: Additional file 1: Table S1. — FGE isolated from Expi293™ cell culture contains both copper and calcium as measured by ICP-MS. Figure S1. Intracellular FGE levels can be determined by flow cytometry or ELISA. Figure S2. The effect of copper(II) sulfate addition on conversion is not replicated by the addition of other metal cofactors. (DOCX 641 kb) [file 12896_2016_254_MOESM1_ESM.docx]

Additional file 1

# Generating aldehyde-tagged antibodies with high titers and high formylglycine yields by supplementing culture media with copper(II)

Dona York,‡ Jeanne Baker,† Patrick G. Holder,† Lesley C. Jones,† Penelope M. Drake,† Robyn M. Barfield,† Gregory T. Bleck,‡ David Rabuka†*

† Catalent Pharma Solutions

5703 Hollis Street, Emeryville, CA 94608

‡ Catalent Pharma Solutions

726 Heartland Trail, Madison, WI 53717

Table S1. FGE isolated from Expi293^TM^ cell culture contains both copper and calcium as measured by ICP-MS.

| Media Treatment | [Protein] (µM)* | Ca (µg/L) | Ca (µM) | Ca (ratio) | Cu (µg/L) | Cu (uM) | Cu (ratio) | |
| --- | --- | --- | --- | --- | --- | --- | --- | --- |
| No added Cu | 57.2 | 97.59 | 97.4 | **1.70** | 113.6 | 1.79 | **0.03** | |
| +50 µM CuSO4 | 47.3 | 68.97 | 68.83 | **1.46** | 2216.8 | 34.88 | **0.74** | |
| *Protein concentrations were measured using the absorption at 280 nm of stock protein solutions. Extinction coefficients for protein were calculated from the primary sequence of the enzyme using the analysis tools on the ExPASy bioinformatics server (5). Hs-FGE, ε=85,870 M-1cm-1 and MW=40,146 Da | | | | | | | |  |
|  |  |  |  |  |  |  |  |  |

**Figure S1. Intracellular FGE levels can be determined by flow cytometry or ELISA.**

A panel of FGE-expressing clones was assessed for FGE levels by flow cytometry (A) or ELISA (B).

The ELISA results were normalized to total protein as determined by the bicinchoninic acid assay (BCA). ALQ denotes samples where FGE levels were above the limit of quantitation in the ELISA. Overall, the results from the two assays showed similar trends in terms of relative FGE levels among the clones.

**Figure S2. The effect of copper(II) sulfate addition on conversion is not replicated by the addition of other metal cofactors.** In order to test the specificity of the observed copper effect on fGly yields, we tried adding various metal ions that are common enzyme cofactors, including FeSO4 (A), MnCl2 (B), and ZnCl2 (C). The metals were added at the indicated concentrations on day 0. Cells were supplemented with 10% Efficient Feed C on Day 3 or 4, as well as 6, and 8. Media was harvested at day 10 (C), 12 (B), or 14 (A), titers (indicated by the numbers atop bars, mg/L) were assessed by ELISA, and conversion was determined by mass spectrometry.

REFERENCES

1. MacLean, B., Tomazela, D. M., Shulman, N., Chambers, M., Finney, G. L., Frewen, B., Kern, R., Tabb, D. L., Liebler, D. C., and MacCoss, M. J. (2010) Skyline: an open source document editor for creating and analyzing targeted proteomics experiments. *Bioinformatics*. **26**, 966–968 10.1093/bioinformatics/btq054

3. Bleck, G. T., York, D., and Collins, I. (2010) Fusion antibodies. [online] http://www.google.com/patents/US20100227394 (Accessed April 30, 2015)

4. Bremmel, R. D., Miller, L. U., and Bleck, G. T. (2008) Host Cells Containing Multiple Integrating Vectors. [online] http://www.google.com/patents/US20080286779 (Accessed April 30, 2015)
